# A unique case of lymphoepithelioma-like HCC with osteoclast-like giant cells: CT imaging features with pathologic correlations

**DOI:** 10.1007/s12328-023-01871-1

**Published:** 2023-10-21

**Authors:** Barbara Frittoli, Anna Castaldo, Marika Santarsiere, Raffaele Ascione, Giulia Tanzi, Andrea Ponsiglione, Gian Luca Baiocchi, Luigi Grazioli

**Affiliations:** 1 Department of Radiology, ASST Spedali Civili, Brescia, Italy; 2 Department of Advanced Biomedical Sciences, University of Naples Federico II, Via Pansini 5, 80131 Naples, Italy; 3 Department of Pathology, ASST Cremona, Cremona, Italy; 4 Department of Clinical and Experimental Sciences, Surgical Clinic, University of Brescia, Brescia, Italy; 5 Department of Surgery, ASST Cremona, Cremona, Italy

**Keywords:** Hepatocellular carcinoma (HCC), Variants, Computed tomography, Histopathology

## Abstract

Hepatocellular carcinoma (HCC) is the most common primary malignancy of the liver, with several histological variants being reported in literature. Hereby, we describe a case of a 77-year-old man with chronic liver disease referred to our department for performing a computed tomography (CT) due to a liver mass discovered at an abdominal ultrasound follow-up. At CT, a large, ill-defined lesion in the third hepatic segment was detected, characterized by progressive and delayed enhancement with minimal retraction of the hepatic capsule, associated with perihepatic adipose tissue inhomogeneity, mimicking a cholangiocarcinoma. At histopathological evaluation, the lesion turned out to be an HCC with lymphoepithelioma-like component and osteoclastic-like giant cells. This report focuses on the clinicopathological and radiological features of this unique case.

## Introduction

Hepatocellular carcinoma (HCC) is the sixth most common cancer and the second leading cause of cancer mortality worldwide, with an incidence rate highest in Eastern Asia and sub-Saharan Africa [1].

Computed tomography (CT) and magnetic resonance imaging (MRI) allow a definitive diagnosis when HCC presents typical features such as hypervascularization in the arterial phase, the hypovascular appearance to the surrounding parenchyma in the portal and late phase, the presence of pseudocapsule as well as dimensional growth at follow-up [2]. However, some HCCs may show an atypical behavior that can make the differential diagnosis challenging; this behavior may be linked to the histology and biology of the different HCC subtypes. Several histological variants of HCC [3] have been reported in literature. Among these, lymphoepithelioma-like HCC (LEL-HCC) represents a rare undifferentiated epithelial carcinoma characterized by massive lymphoid infiltration that frequently develops in cirrhotic livers, strongly related to hepatitis B or C, with a better prognosis compared to classic HCC form. There are no specific clinical presentations, and imaging findings are etherogeneous [4].

HCC with Osteoclast-like giant cells (OGCs) is a rare variant of HCC, with only a few cases reported. This variant may also occur in cirrhotic liver but seems more aggressive, frequently presenting with lung and bone metastases at diagnosis. Main presentation symptoms include abdominal pain, weight loss, general fatigue and jaundice. Imaging findings are not specific and the cases reported show the same radiological characteristics of HCC [5].

Hereby, we report a unique case of LEL-HCC mixed with OGCs in a 77-year-old man with chronic liver disease, as assessed by CT.

## Case report

A 77-year-old man with metabolic liver disease and previous left hemicolectomy for pT4N0 colon cancer 6 years before, without adjuvant chemotherapy, was referred to our institution for further investigation on a liver lesion detected through a follow-up ultrasound examination. The patient was affected by several comorbidities such as metabolic syndrome, uncontrolled diabetes, hypertension, dyslipidemia, and hyperuricemia. Routinary biochemical blood tests were unremarkable and serological markers for hepatitis B and C virus were not detected.

**Fig. 1:**
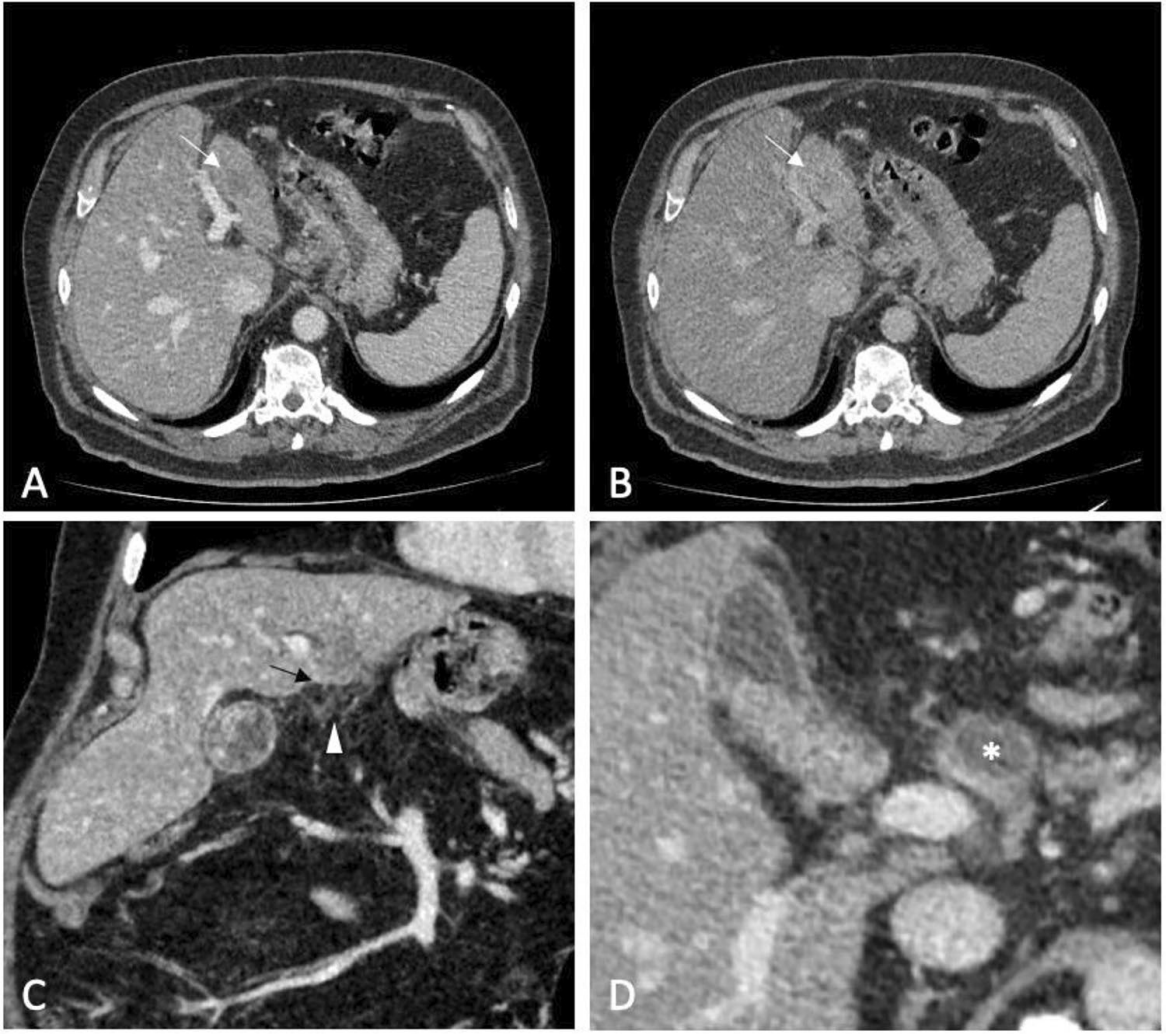
Axial contrast-enhanced CT images in portal (**A**) and equilibrium (**B**) phases showing a lesion in the III hepatic segment with progressive and delayed enhancement (white arrows). Minimal retraction of the capsule (black arrow) and perihepatic adipose tissue inhomogeneity (arrowhead) are visible in coronal view (**C**). Axial contrast-enhanced CT image in portal phase (**D**) also shows a hepatic hilar lymphadenopathy adjacent to the hepatic artery and characterized by a large central necrotic component (asterisk)

Contrast-enhanced CT (CE-CT) was performed to confirm the presence of a large, ill-defined lesion located in the third hepatic segment. The lesion showed progressive and delayed enhancement with minimal retraction of the hepatic capsule associated with perihepatic adipose tissue inhomogeneity (Fig. 1A–C). Hilar and common hepatic artery lymphadenopathies were also detected, reaching approximately 3 cm maximum in diameter (Fig. 1D).

Several considerations were made for the differential diagnosis: the presence of a single large lesion and the time elapsed since the diagnosis of primary colon cancer made the diagnosis of primary liver cancer rather than metastasis more likely. The history of a chronic liver disease combined with the imaging features—with special reference to nodal metastases—pointed towards a cholangiocarcinoma.

**Fig. 2:**
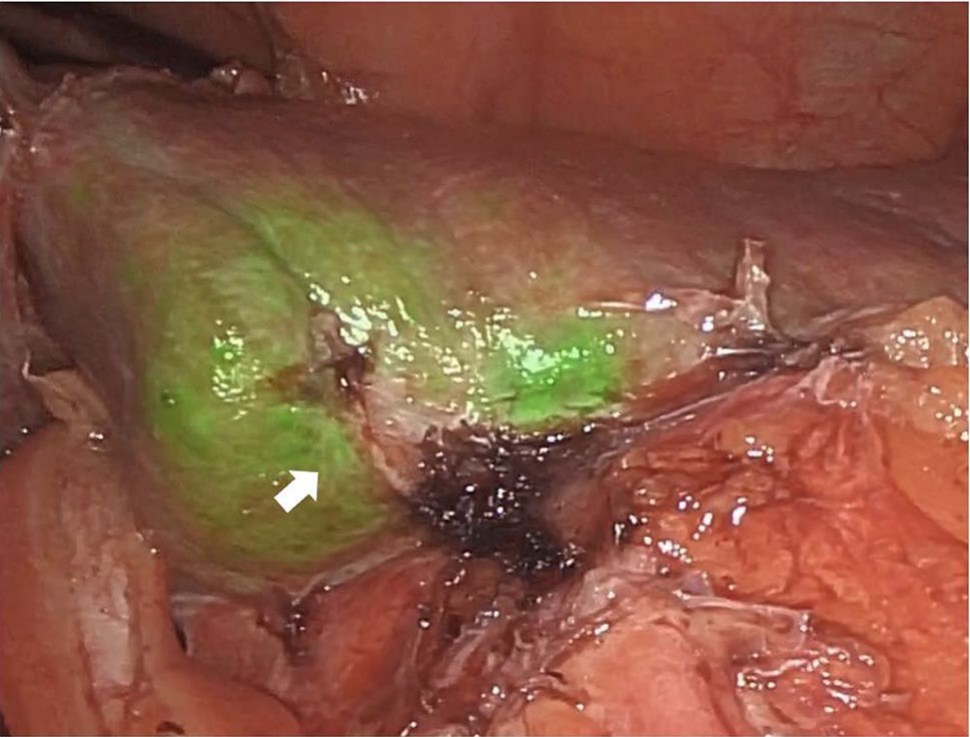
Intraoperative fluorescence of the area under examination retaining indocyanine green, with a zone of retraction on the surrounding tissues (arrow)

After a multidisciplinary team discussion, surgery was indicated. The day preceding the intervention, 2 mL of 2.5 mg/mL indocyanine green was injected intravenously. Surgical approach was performed by laparoscopy; the intraoperative fluorescence of the tumor was typical for HCC [6] (Fig. 2). Left lobectomy was performed, without the need of pedicula clamping and transfusions. Extended lymphadenectomy was also performed, including nodal stations 12a, 12 b, 12 p, 8a, 8b, 8p, 7 and 9 [7].

Macroscopically, a 3.8 × 2.5 × 1 cm grey–white hard tumour mass with yellowish areas and micronodular appearance was evident in the resected section. The surgical margin was free of tumor tissue, and no lymphovascular invasion or capsule involvement was identified.

**Fig. 3:**
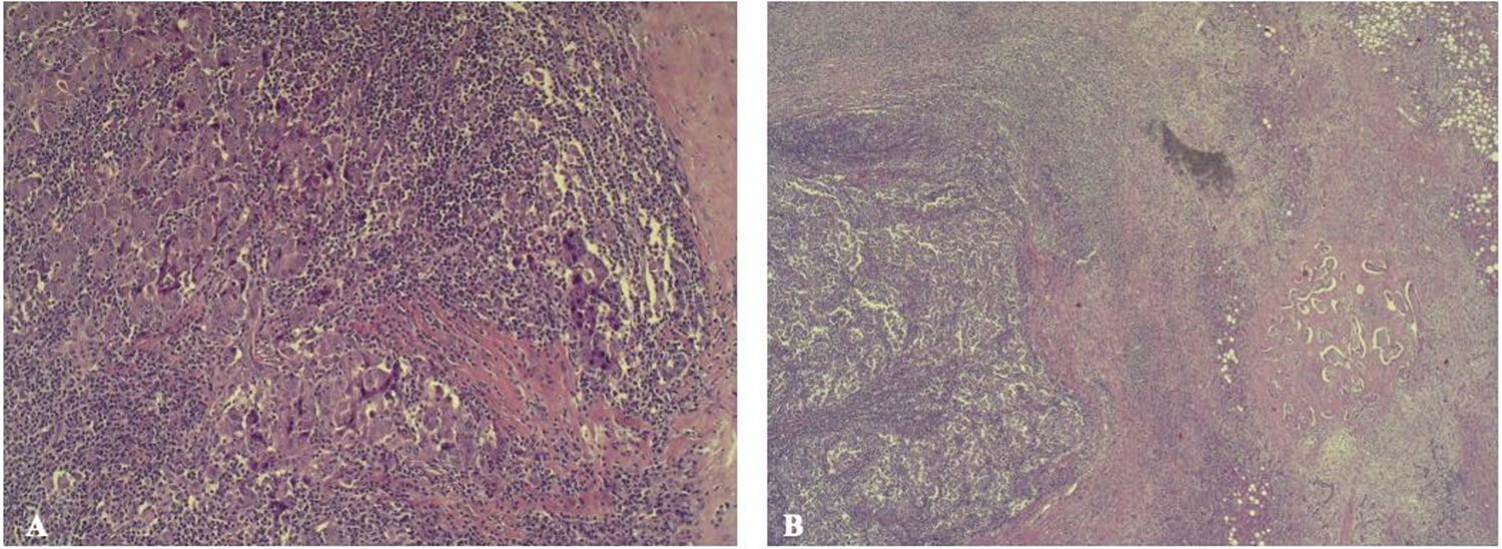
Hematoxylin–eosin stain with 10× (**A**) and 2.5× (**B**) magnification, showing epithelial proliferation in solid chords and aggregates consisting of medium-sized cells with a rounded hyperchromatic nucleus, relatively large cytoplasm, and a mixed lymphoepithelioma-like inflammatory population with some Osteoclast-like giant cells

The lesion showed epithelial proliferation in solid chords and aggregates consisting of medium-sized cells with a rounded hyperchromatic nucleus, relatively large cytoplasm, and a mixed lymphoepithelioma-like inflammatory population with some OGCs (Fig. 3).

**Fig. 4:**
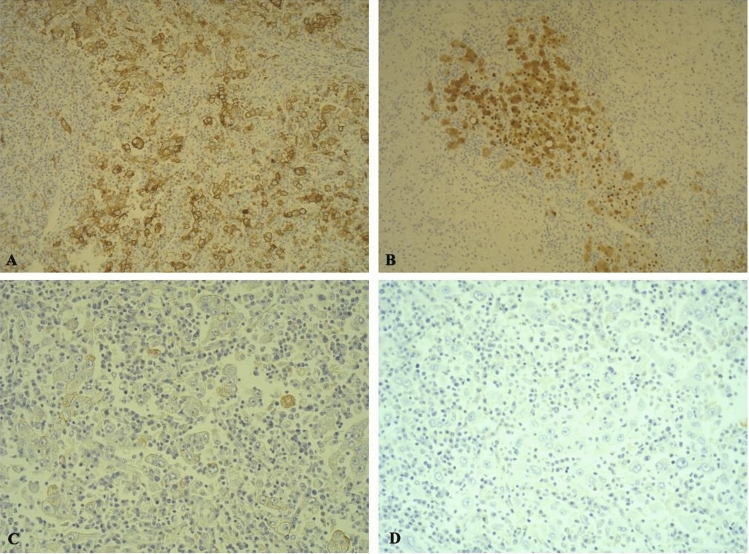
Immunohistochemical staining respectively with CD10 (**A**) and Arginase (**B**) antibodies at 10 × magnification and Cytokeratin 7 antibody at 20 × magnification (**C**), while negative for CK19 at 20 × magnification (**D**)

Immunohistochemical analysis (Fig. 4) revealed that tumoral cells were positive for CD 10, Glypican3, CK 7, TFF-1, and arginase; EBER ISH 1-2 excluded EBV infection.

Pathology also confirmed chronic liver disease demonstrating steatohepatitis with low-grade fibrosis. Furthermore, one node (level 8b) tested positive for localization of hepatocellular carcinoma.

These features supported the diagnosis of a poorly differentiated HCC with lymphoepithelioma-like components and osteoclastic-like giant cells in a pT1b pN1 stage.

**Fig. 5:**
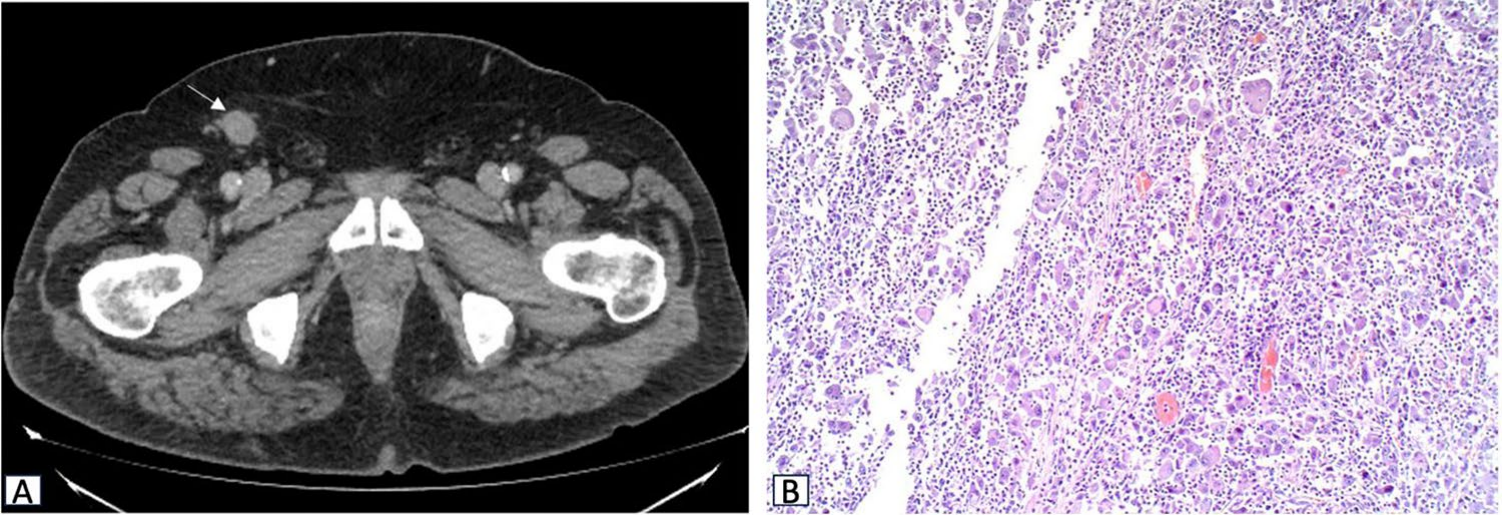
Axial contrast-enhanced CT image in portal phase showing a right inguinal lymphadenopathy (arrow in **A**), with immunophenotypic features similar to the primary liver lesion (10 × magnification, **B**)

The patient did not suffer from any complications after surgery, and a follow-up strategy was preferred rather than an adjuvant chemotherapy treatment. However, at 6-month follow-up CE-CT showed a right inguinal lymphadenopathy, that was surgically removed and pathologically confirmed as metastatic (Fig. 5).

## Discussion

HCC is the most common primary malignancy of the liver, with cirrhosis from alcohol and viral aetiologies representing the main risk factor [1].

Clinical presentation is variable, and it is often detected during screening programs performed in patients with cirrhosis or other liver disorders. Otherwise, constitutional symptoms, jaundice, portal hypertension signs, and haemorrhage may represent the first signs of disease.

Imaging allows a definitive diagnosis avoiding biopsy when the tumor presents with typical features. CT and MRI can easily detect HCC by demonstrating the presence of major criteria established by the guidelines of the American Association for the Study of Liver Diseases (AASLD) and the Liver Imaging Reporting and Data System (LI-RADS) [8, 9]. These criteria include hypervascularization in the arterial phase (“wash-in”), the hypovascular appearance to the surrounding parenchyma in the portal and late phase (“wash-out”), the presence of pseudocapsule and dimensional growth. MRI with hepatobiliary contrast medium has higher sensitivity and specificity for diagnosing HCC since it can outline other ancillary findings such as intracellular fat, hyperintensity in T2, restricted diffusion in DWI sequences, and hypointensity in the hepatobiliary phase [2].

However, some HCCs do not show these classical features at imaging, while presenting an atypical behavior which makes differential diagnosis challenging. Several histologically and biologically distinct subtypes of HCC have previously been described, and although the number of cases reported in literature is limited, they must be considered for the differential diagnosis [10, 11].

Clear Cell HCC (CCHCC) represents a variant characterized by glycogen and lipid in the cytoplasm of tumor cells, divided into a diffuse pattern with more than 50% clear cells and a focal pattern with about 30% [10]. These features can translate into increased echogenicity on US, reduced attenuation on unenhanced CT and a signal drop on opposed-phase T1-weighted MRI [3].

Another rare variant of HCC is represented by the medullary-like one [12]. Besides liver, medullary tumors can arise in many locations like thyroid, breast, pancreas, stomach, and colon. It can show a syncytial growth pattern, high-grade nuclear features, necrosis, infiltrating lymphocytes and plasma cells.

Combined hepatocellular-cholangiocarcinoma is a rare type that shows phenotypic features suggestive of hepatocytic and biliary differentiation. The CT and MRI post-contrast characteristics depend on the proportions of tumor components [3].

An aggressive variant of HCC is represented by the sarcomatoid one, characterized by the predominance of spindle cells. These poorly differentiated cells grow fast, determining central necrosis or haemorrhage, which results in a mostly peripheral enhancement with an ipondense central portion on contrast imaging [3].

Two of the rarest histological variants are LEL- and OGC-HCCs. LEL-HCC is a particular form of undifferentiated epithelial carcinoma characterized by massive lymphoid infiltration, recently recognized by The World Health Organization as a variant of HCC [4]. LEL-HCC was related to cirrhosis in 46% of the cases, with underlying hepatitis B and C virus, respectively, in 40% and 34% [4]. Most patients were asymptomatic, while a few have showed right upper abdominal pain or symptoms like chronic cholangitis [13, 14]; alpha-fetoprotein may be elevated [14].

Imaging findings of LELC are not well known and could mimic HCC. Solinas et al. described two different imaging patterns [15]. At CT and MRI, the first one showed homogeneous enhancement in the arterial phase and subsequent wash-out in the portal phase, as for typical HCC. In the second case, CT scan did not show arterial enhancement but just a peripheral rim enhancement associated with a T2 hyperintensity at MRI. Macroscopically, the tumour is generally described as a gross, well-circumscribed, grey–yellow–white and soft solid mass with variable capsule. Microscopically it is poorly differentiated, consisting of atypical cells with syncytial cytoplasm, nuclei with prominent nucleoli and abundant lymphocytes infiltration [4].

Labgaa et al. [4] reported pankeratin (e.g., AE1/AE3), low molecular-weight keratin (e.g., K8/18), EMA and HepPar-1 as its principal immunohistochemical markers. The massive lymphocyte population was composed of CD3 + T-cells with CD20 + B-cells and a mixture of CD4 and CD8 + T-cells. The prognosis reported seems to be better than the conventional HCC (5-year survival of 67%), although the clinical outcomes are variable [16].

OGC is an extremely rare variant of HCC: only 14 cases have been reported till today [5], with the first one described in 1980 by Munoz et al. [17]. Typically, this tumor arises in a cirrhotic liver, and both lung and vertebral metastases have been observed at the time of diagnosis. According to Gielen et al. [5] the most reported clinical symptoms at presentation are abdominal pain, weight loss, general fatigue and jaundice. Imaging findings are not specific, and mostly they show the same radiological features of HCC. In a previous case report by Rudloff et al., an HCC with OGCs was detected at CE-CT as a 6 cm heterogeneous solid mass, subsequently confirmed on MRI as a relatively well-circumscribed lesion with multiple fluid-like signals areas, suggestive of necrosis or cystic components [18]. At pathology, the tumor generally results in a white to brown mass with multiple areas of necrosis and haemorrhage. Microscopically, mononuclear cell (MNC) and less frequently pleomorphic large cells (PLC) may be observed [17, 18]. The immunochemistry profile described by Rudloff and Gielen [5, 18] showed CD68 + and vimentin + osteoclast-like giant cells, with low positivity for alpha-1-antitrypsin and alpha-1-chymotrypsin, while the infiltrating MNCs showed high positivity for alpha-1-antitrypsin and alpha1-chymotrypsin. Neoplastic population showed positivity for citokeratine 7 too, that is present in hepatic progenitor cells (HPCs) and in cholangiocytes but not in normal hepatocytes. HCCs with OGCs usually present an aggressive clinical and biological behavior with an unfavorable prognosis even after resection.

In our case, several considerations were made for the differential diagnosis, which included HCC and its variants, cholangiocarcinoma and metastasis [19]. CECT confirmed the presence of a large, ill-defined mass located in the left hepatic lobe which did not show typical imaging features of HCC. A benign nature was excluded, considering the new onset, the atypical enhancement pattern, the minimal capsule retraction as well as the presence of hilar and common hepatic artery lymphadenopathies. At first, we hypothesized a diagnosis of cholangiocarcinoma due to the progressive and delayed enhancement; furthermore, the association of minimal hepatic capsular retraction, enlarged hilar lymph nodes and a history of chronic liver disease sustained our hypothesis. Generally, mass-forming intrahepatic cholangiocarcinoma (MF-iCCA) is less common than HCC and cHCC-CCA in the cirrhotic liver [20]. Typically, it appears as an ill-defined mass with rim-like enhancement in the arterial phase and progressive centripetal enhancement in the subsequent phases [21]. However, in cirrhotic liver, atypical global arterial enhancement with wash-out may be more commonly seen in MF-iCCA than in those arising from non-cirrhotic patients [21]. Furthermore, peritumoral biliary dilation may be useful to distinguish MF-iCCA from HCC in the non-cirrothic liver, but it may be absent in tumour arising in cirrhosis [22].

Metastasis was another critical differential diagnosis in our patient since he suffered colon cancer 6 years before. Metastases are relatively uncommon in cirrhotic liver, probably due to alterations of hemodynamics and microstructural environment in the liver [23]. When metastatic cancers are found in cirrhotic liver, they usually show rim-like arterial phase hyperenhancement (APHE) and can be categorized as non-HCC malignancy according to the imaging criteria of LI-RADS [2]. However, when metastases occur in cirrhotic liver, they may demonstrate both non-rim APHE and wash-out appearance, and consequently be misdiagnosed as HCC. In our case, the presence of a single large lesion in a cirrhotic liver and the time interval since the colon cancer diagnosis favored a primary liver lesion.

At pathology examination, the mass turned out to be characterized by two histological variants of HCC, respectively, the LEL and OGC ones. To the best of our knowledge, these variants, even rare when considered individually, have never been never described together in the literature.
